# The Effects of Psychotherapist's and Clients' Interpersonal Behaviors during a First Simulated Session: A Lab Study Investigating Client Satisfaction

**DOI:** 10.3389/fpsyg.2017.01868

**Published:** 2017-10-31

**Authors:** François Moors, Emmanuelle Zech

**Affiliations:** Psychological Sciences Research Institute, Université catholique de Louvain, Louvain-la-Neuve, Belgium

**Keywords:** interpersonal behaviors, complementarity, psychotherapy, agency, warm, satisfaction, drop-out

## Abstract

The purpose of this study was to examine the effects of psychotherapists' behaviors during a first simulated therapy session on clients' satisfaction, including their intention to pursue or drop out from therapy. The importance of psychotherapists' warmth on clients' satisfaction was examined to check previous findings stressing this determining factor. Examining the role of warm behaviors is however insufficient according to the interpersonal perspective. We therefore tested the role of the psychotherapist's agentic behaviors since only a few studies provide contradictory results about the role of this interpersonal dimension on clients' satisfaction and how it is influenced by matching up client and therapist's profiles. To test our hypotheses and control for alternative therapy-related explanatory variables, we used different videos as experimental conditions manipulating the therapist's behaviors. Seventy-five participants had to imagine themselves as potential clients arriving for a first therapy session. They successively watched a role-playing therapist behaving according to five randomized interpersonal profiles. Results confirmed that warmth was a major dimension predicting client satisfaction. They revealed that agency was also a determinant of client satisfaction and that its effects depended on the client's own interpersonal agentic profile. Dominant clients were found to be more satisfied with the dominant psychotherapist than the submissive one while submissive clients preferred only the warm psychotherapist. These findings are discussed and suggest that therapists may need to be flexible and adapt their behaviors according to their client's interpersonal profile to increase their client satisfaction and decrease drop outs.

## Introduction

In human relationships, interlocutors form an impression at the beginning of a new relationship (e.g., Fiske and Neuberg, 1990; Bodenhausen et al., 2012). Whether an interlocutor leaves a good or a bad impression has consequences on whether the relationship will continue or not (e.g., Human et al., 2013). In the present paper, we examine whether this is also the case in the context of a new professional psychotherapeutic relationship and thus whether the first contact with a psychotherapist will be decisive for client satisfaction and the continuation of the relationship. It is important to encourage a high level of client satisfaction within a therapeutic relationship because this influences therapeutic outcomes not only by decreasing the presence of symptoms (e.g., Conte et al., 1995) but also by increasing treatment compliance (Vermeire et al., 2001). More fundamentally, client dissatisfaction may be a predictor of dropouts (Bados et al., 2007; Samstag et al., 2008), which are frequent in helping relationships. Indeed, meta-analyzes show attrition rates between 35% (Roos and Werbart, 2013) and 47% (Wierzbicki and Pekarik, 1993). Among the factors influencing client satisfaction, it is likely that how the psychotherapist behaves at first sight, i.e., the first impression that he or she makes, will have a direct impact on client satisfaction. However, few studies have examined this question (Conte et al., 1995; Ogrodniczuk et al., 2007).

Previous psychotherapy research has examined several factors explaining satisfaction with psychotherapists, such as gender (e.g., Zlotnick et al., 1998) or level of experience (e.g., Baekeland and Lundwall, 1975). It has also been established that professional helpers' warm behaviors positively influence general client satisfaction (e.g., Ryan and Moses, 1979; Thompson et al., 2011). This has consistently been shown in recent meta-analyzes: helpers' warm behaviors, as part of the helper's unconditional positive regard, are essential for therapeutic effectiveness. Throughout therapy sessions, warm behaviors improve the therapeutic alliance, decrease the risk of dropout, and increase therapeutic outcomes (e.g., Farber and Doolin, 2011; Roos and Werbart, 2013). On the contrary, helpers perceived as cold or distant by their clients have poorer therapeutic results (Hersoug et al., 2009). It therefore appears that therapists' warmth is an essential condition for increasing client satisfaction, enhancing the therapeutic alliance and avoiding the risk of dropout.

However, it appears that warmth is not always sufficient to produce overall client satisfaction. For instance, a review by Ackerman and Hilsenroth (2003) showed that not only is it important to be warm, but it is also useful for psychotherapists to be involved and active in the therapy. Conte et al. (1995) also emphasized that psychotherapists perceived as too inactive would generate a high level of dissatisfaction among their clients. Finally, it has been shown that active listening or giving advice rather than only giving verbal and nonverbal acknowledgments increase client satisfaction (Weger et al., 2014). These results suggest that, in addition to warm behaviors, psychotherapists should also be perceived as assertive.

**Figure 1:**
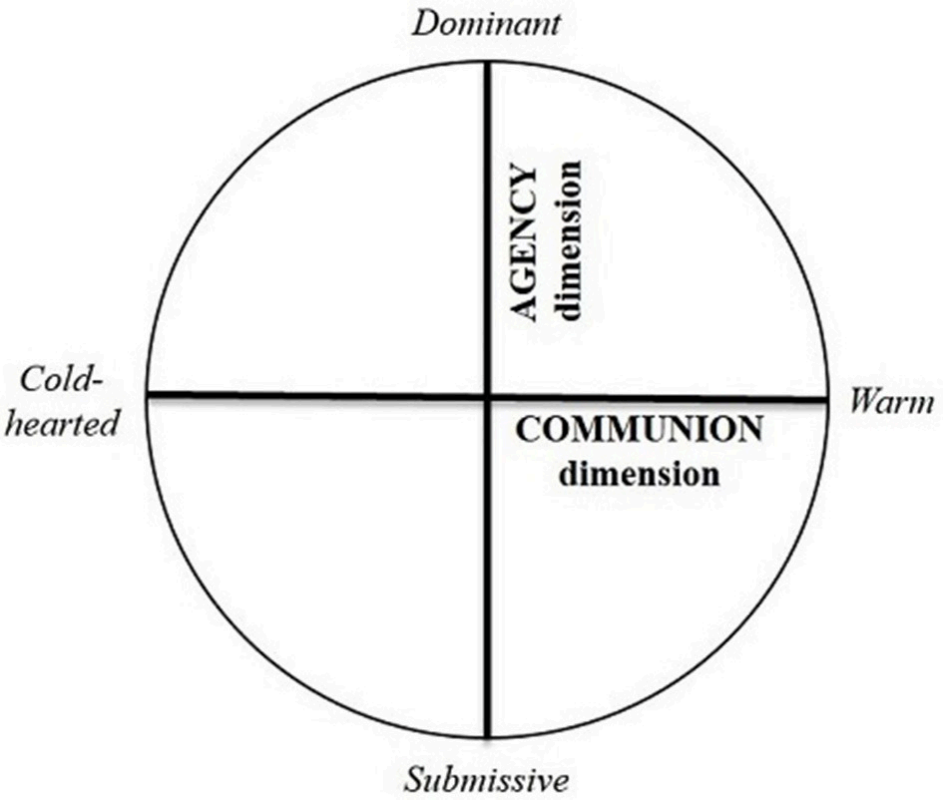
The circumplex model. Presentation of the Circumplex Model (adapted from Wiggins, 1995), with the two interpersonal dimensions and the labels of the extremes.

Explaining initial client satisfaction depending on psychotherapists' behaviors during a first session therefore requires a model that conceptualizes all the interpersonal behaviors at play. In social and personality psychology, the circumplex model (Fournier et al., 2011) was designed to explain interpersonal behaviors and relationships (Strack and Horowitz, 2011). This model has been widely validated empirically (see Gurtman, 2011). It conceptualizes interpersonal behaviors, based on two dimensions (see Figure 1). While warm behaviors are characteristic of an interpersonal dimension named the *warmth, affiliative or communion dimension*, agentic behaviors are defined by a second interpersonal dimension called the *dominance, control or agency dimension* (Fournier et al., 2011). The first dimension considers the ability with which the person begins an interaction with others (Fournier et al., 2011). On one end of this dimension, the specific behaviors are the warm or approach behaviors. At the opposite end are the cold, distant and even hostile or indifferent behaviors (Horowitz et al., 2006). The second dimension concerns the controlling and autonomous behaviors people exert on their environment (Fournier et al., 2011). Thus, the characteristic behaviors at one end of this dimension are dominant behaviors, while nonassertive or submissive behaviors are at the other end. The impact of interpersonal behaviors has largely been examined in the context of common interpersonal relationships. In particular, warmer or affiliative and more agentic or competent persons are generally more appreciated than cold and unassertive persons (O'Connor, 2011). However, the impact of these two dimensions on client satisfaction has not yet been examined during a first therapy session. Because of the high consistency regarding the importance of warm behaviors in the psychotherapy literature, we first postulated that there would be a major effect of the *communion* dimension on client satisfaction. Specifically, a warm therapist would be more satisfying than a cold therapist. Our second hypothesis relates to the *agency* dimension. Even if the psychotherapy literature seems to be divided about the importance of being directive or nondirective as psychotherapist (Cain, 2013), several studies highlight the benefits of being active (e.g., Conte et al., 1995; Ackerman and Hilsenroth, 2003; Weger et al., 2014). We therefore postulated that a dominant therapist would be more satisfying than a submissive one.

Besides the impacts of both dimensions, the general impression about an interlocutor has been found to be moderated by the matching between both individuals' interpersonal characteristics (Kiesler, 1996). Indeed, the interpersonal literature has found that when two persons are interacting, it is necessary to consider the respective interpersonal profile of each interlocutor to determine the most satisfactory interactional matches (Kiesler, 1983; Sadler and Woody, 2003). But the literature is unclear about the most satisfying matchings. Two main theoretical perspectives define these matchings. One predicts that complementary behaviors lead to satisfaction. On the contrary, the other postulates the importance of similarity.

From the interpersonal perspective, the concept of complementarity defines the satisfying interactions between two interlocutors (Kiesler, 1996). In such a situation, two individuals' behaviors are judged as complementary, and therefore more satisfying, when one person exhibits dominant and the other submissive behaviors and when they both exhibit either warm, or cold behaviors. Many studies in different kinds of relationships have confirmed that the more complementarity there is between two persons, the more these persons will be satisfied with the relationship (e.g., Tracey, 2005; Sadler et al., 2011). In the context of professional helping relationships, few studies have examined whether complementarity between therapist and a client profiles has a positive impact on client satisfaction during a first session. Nevertheless, several studies have confirmed the benefits of psychotherapist-client complementarity on several variables. For instance, Kiesler and Watkins (1989) studied the impact of complementarity on the therapeutic alliance. They asked 36 dyads of therapists-clients to complete a set of questionnaires after the third therapy session. Their results showed that the stronger the complementarity for the cold side of the communion dimension, the more both patient and therapist perceived a strong therapeutic alliance. Tracey et al. (1999) analyzed the evolution of complementarity over six therapeutic sessions. By studying 20 follow-ups of cognitive behavioral therapy, they emphasized that the dyads obtaining the best therapeutic outcomes were those that showed initial high complementarity, decreasing in the middle sessions and increasing at the end of therapy. Finally, Svartberg and Stiles (1992) showed that, during a 20-session dynamic psychotherapy, complementarity was a better predictor of client change than the competence of the therapist.

The literature on synchrony presents an alternative hypothesis related to how interpersonal matchings are most satisfactory or explaining how people interact most effectively. Several studies showed that emergent behavioral coordination can occur during joint action between two protagonists. When at least two individuals interact to change their immediate environment, they show a coordination of or a similarity in their behaviors (Knoblich et al., 2011). In that kind of situation, spontaneous similar behaviors occur in both interlocutors (Knoblich et al., 2011). This synchronization can include, for instance eye movements (Richardson et al., 2007), hand movements (Wallot et al., 2016), or even physiological aspects (Mønster et al., 2016). This kind of synchronization generally impacts the product outcomes and interlocutors' satisfaction positively (Wallot et al., 2016). Transposed to the psychotherapeutic relationship, this literature could suggest that if therapists present interpersonal behaviors that are similar to their clients', this might lead to higher client satisfaction. A few studies have also shown that nonverbal synchrony has positive impacts on the psychotherapeutic relationship (e.g., Ramseyer and Tschacher, 2011).

Given the literature about the potential impact of the matching between interlocutors' interpersonal behaviors, it is likely that clients' interpersonal profiles will also determine their level of satisfaction depending on its match with the therapist's profile. Because both complementarity and similarity have been found to have a positive impact on the relationship, it is difficult to predict whether a client will be more satisfied by a complementary or a similar therapist. Our third hypothesis postulated that clients' interpersonal profile would determine their satisfaction, but the direction of this effect remained exploratory.

It is important to note that, both in terms of complementarity and of synchrony in a psychotherapeutic context, these different studies were designed to observe the effects of complementary or similar behaviors over a long period of time, in other words, during several sessions or interactions. The purpose of our research is to study client satisfaction during an initial session as a predictor of drop-out, a faster phenomenon, linked to the interlocutors' first impressions. In other words, previous studies did not specifically examine the impact of the therapist's behaviors on client satisfaction during the first session, nor whether this first impression influences drop out. In fact, in real therapeutic settings, clinical variables such as the therapeutic alliance, the types of client difficulties or disorders, the type of therapeutic approach or protocol, will be additional major intertwined determinants of client satisfaction. While ecological studies take account of the complexity of clinical phenomena holistically, they do not allow to isolate the specific effect related to the therapist's interpersonal behaviors and interactions with the client's interpersonal profile. Also, such studies do not allow to estimate whether a client would be more satisfied with one or another therapist, or more specifically with the types of behaviors that the therapist presents, nor whether the client's own interpersonal profile interacts with the therapist's interpersonal profile. Indeed, in such studies, clients are not presented with different therapists and are thus not able to compare and choose between them according to their satisfaction since they are allocated to just one interlocutor.

Therefore, our clinical question “which psychotherapist behaviors influence client satisfaction during a first session of psychotherapy?” will find answers only in a controlled setting. To determine the relative contribution of warm and agentic behaviors on client satisfaction, as well as the type of satisfactory interpersonal matches between a client and a therapist, it is thus necessary to create and compare such conditions in a controlled environment from which most alternative explanatory variables are excluded. For this purpose, we created an original design in which a first therapeutic session with a therapist was reproduced in a lab context. The experiment uses simulated interactions with a psychotherapist in the context of a very common life experience: the breaking up of a romantic relationship. The lab context allowed to generate different interpersonal profiles for the therapist's behaviors.

## Method

### Participants and recruitment

Seventy-five (52 women and 23 men) French-speaking adults (*M*_age_ = 25.35 years old, *SD*_age_ = 10.95 years old) participated in the study, approved by the institutional ethical committee. All of them were Caucasian and had at least 12 years of education. The majority were students (81.33%).

The participants were recruited via notices on the university campus. They could contact the researchers by email or phone. They received 6€ for their participation in this study.

### Material and procedure

**Figure 2:**
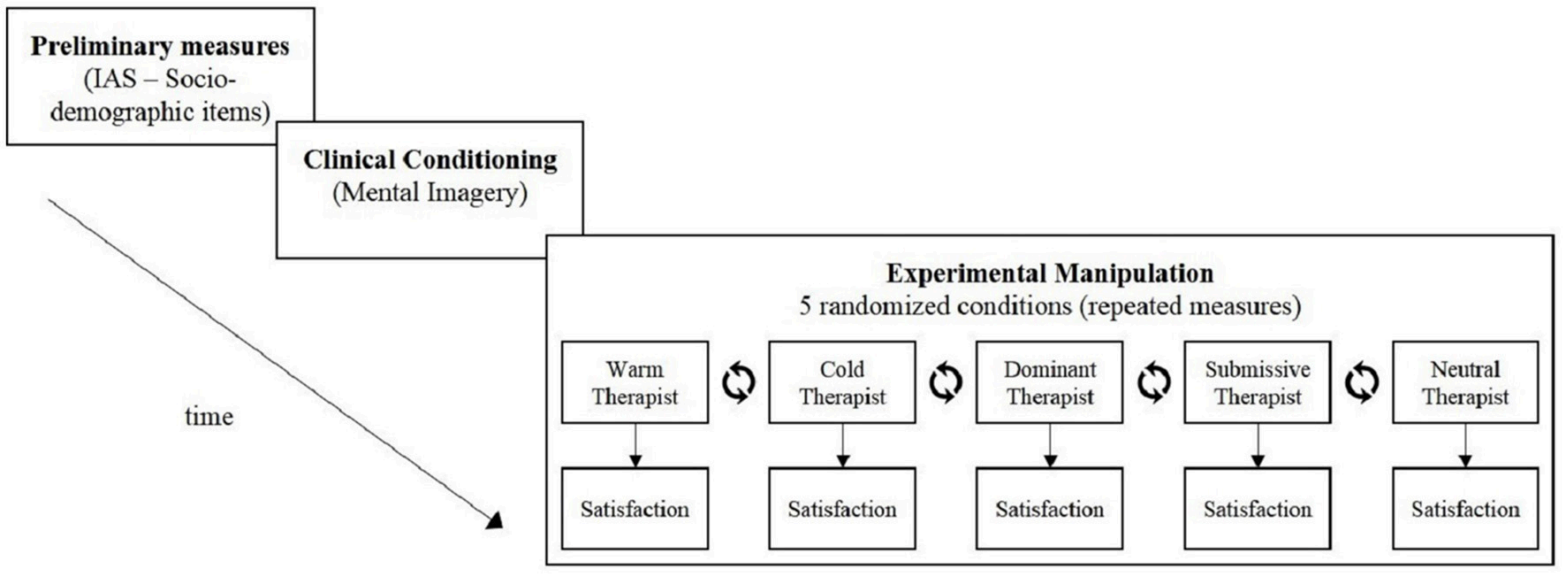
Time-line of the experimental procedure. Presentation of three main steps of the experimental procedure. Each participant had to follow these steps and was confronted to the five experimental conditions manipulating the therapist's interpersonal profile.

The use of a lab context in which the procedure was standardized, combined with precautions to decrease artificiality as described below, allowed to isolate and control the determinants of clients' satisfaction. Each participant followed three steps (see Figure 2).

#### Step 1: preliminary measures

Before coming to the lab, participants filled in an online questionnaire. This questionnaire comprised the Interpersonal Adjective Scales (IAS, Wiggins, 1995) and the socio-demographic items, described below.

#### Step 2: clinical conditioning

About a week after the questionnaire had been filled in, participants were invited to the lab. Before the beginning of the experimental manipulation, participants were exposed to clinical conditioning by mental imagery (Hackmann et al., 2011); this consisted of listening to a 6 min audio recording. Participants were asked to imagine being a client meeting with a psychotherapist. This client was presented as being in a situation of romantic break-up, with different consequences (for instance, social isolation, sleeping problems, or unpleasant affects). The situation of a romantic break-up was chosen because it is commonly experienced and participants could easily imagine being in such a situation, thus ensuring the least possible artificiality of the clinical situation.

After describing the different social and psychological difficulties related to this romantic break-up, the scenario ended with the following sentence: “Imagine that, because of these difficulties, you decide to meet a psychotherapist to find a solution. Imagine you made an appointment with a psychotherapist, David Fisher. Now, imagine that you are in the corridor leading to his office and you are ready to knock on the door”. After hearing this last sentence, participants were placed in front of a large screen (80 × 50 inches), for step 3.

#### Step 3: experimental manipulation

After the clinical conditioning, participants were invited to meet a psychotherapist “fictitiously” in the sense that when they were in front of the big screen, a video was displayed. This video began with a scene viewed as if through the eyes of someone walking along a corridor then a hand knocking on a door marked “David Fisher—Psychotherapist.” Throughout this video, the person (representing the client) sits in front of the psychotherapist. Then the consultation begins in the form of a dialog between psychotherapist and client, lasting approximately 6 min. To heighten ecological quality and decrease artificiality, the whole video was made using the first person and filmed in such a way that participants (below named clients) viewing could have the impression that they were the person walking down the corridor, knocking on the door and speaking with the psychotherapist; the psychotherapist was shown life size, given the height of the screen.

Five experimental conditions manipulating the psychotherapist's behaviors of communion and agency were created (Psychotherapist Conditions). Each experimental condition consisted of a video showing a psychotherapist with verbal and nonverbal behaviors characteristic of a particular interpersonal profile (e.g., Gifford, 1991; Moskowitz, 1994). The five interpersonal psychotherapist profiles were the following: warm vs. cold, dominant vs. submissive, and neutral. These different profiles were constructed using several tools correctly identifying the representative behaviors of each profile correctly: IAS (Wiggins, 1995), Inventory of Interpersonal Problems (Horowitz et al., 2003) and Check List of Psychotherapy Transactions-Revised (Kiesler et al., 1991). Among the different representative behaviors of each profile, the “warm” psychotherapist smiled and reformulated the client's feelings several times; the “cold” psychotherapist tended to take notes instead of looking at the client and used few words, the “dominant” psychotherapist leaned forward, used his hands when he talked and gave many recommendations or explanations; the “submissive” psychotherapist talked quietly, seemed sometimes uncertain and fidgeted with objects such as a pen. The “neutral” psychotherapist's profile was made up of the various characteristics of the other four profiles. The label “neutral” psychotherapist was chosen to represent a psychotherapist who is less extreme both on the agency and the communion dimensions. The labels “warm, cold, dominant, and submissive” psychotherapist were chosen to fit Wiggin's taxonomy (e.g., Wiggins, 1982), Wiggins being a major author of the circumplex theory. This taxonomy is frequently used in the interpersonal research field. Although these labels could be seen as extreme for a psychotherapist, the behaviors for each profile were selected to be suited to the psychotherapeutic setting. Thus, all behaviors, irrespective of profile or label, were considered as possible in a therapeutic setting, even if some behaviors could be more or less likeable.

The five videos were presented to participants in random order. After each presentation, participants completed a questionnaire measuring their satisfaction with the psychotherapist. A distractive task followed to reduce comparisons between the different experimental conditions. This task involved solving a maze in 3 min on an A4 sheet.

Two standardized procedures were used in constructing the videos. First, the contextual elements of the video (i.e., the actor playing the role of the psychotherapist, the consultation office, and the first seconds of the video showing the client's arrival in the consultation office) were identical. Second, the interactions spoken by the client were the same across all experimental conditions. They were also based only on the factual elements introduced in the clinical scenario. In other words, the sentences spoken by the client were as neutral as possible (i.e., relatively short and based on facts described in the clinical conditioning), such that each participant could identify with these typical sentences. In order to help participants imagine being the client, the voice of the actor playing the client was male or female depending on the participant's gender.

### Measures

#### Interpersonal profile

The Interpersonal Adjective Scales (IAS) was used to measure the participants' interpersonal profiles (Wiggins, 1995). This questionnaire is made up of 64 adjectives, each self-evaluated on a Likert scale with eight levels from extremely accurate (1) to extremely inaccurate (8). These evaluations position participants on each of the eight subscales. The scores were standardized (z's scores based on the mean and the standard deviation of our sample) for each subscale. The internal consistency for the subscales was satisfactory (α's between 0.73 and 0.94). The correlation between the two dimensions was not significant in our sample, *r*_(73)_ = −0.01, *p* = 0.908, confirming the orthogonality of the interpersonal dimensions, as postulated by the circumplex model (Fournier et al., 2011; Locke, 2011).

The IAS also makes it possible to locate participants' interpersonal profile on the circumplex by different methods. To test our hypotheses, both communion and agency dimensions for each participant were considered independently. Therefore, two categorizations of the participants were performed, based on the means and standard deviations of each of the two interpersonal dimensions. In addition, we selected participants who were sufficiently representative of the different interpersonal profiles. Thus, participants who were too close to the mean of the dimension according to the sample (i.e., less than 0.5 SD) were excluded from the categorization. Concerning the “communion” dimension, we categorized participants as either “Warm” (*n* = 17), or “Cold” (*n* = 30). A second categorization, based on the “agency” dimension, allowed to categorize participants as either “Dominant” (*n* = 25), or “Submissive” (*n* = 27).

### Satisfaction

Satisfaction is a broad concept determined in several ways (e.g., Speight, 2005) that have found no consensus in the literature. However, it is clear that satisfaction must be operationalized in a multidimensional manner, depending on the research target (Fitzpatrick, 1991). Because this study aims to analyze the influence of interpersonal behaviors in the context of a first therapy session, we chose to operationalize client satisfaction as a subjective evaluation about (1) the session they had just experienced in general, (2) the specific psychotherapist's behaviors and, from a more practical standpoint, (3) the intention to return for a potential second session (as inverted measure of drop out), and (4) an estimation of the amount of money the session may be worth paying for.

First, client satisfaction with the session in general was evaluated with two subscales of the Session Process and Outcome Measures (Hill and Kellems, 2002): the Relationship Scale (4 items, e.g., “In this session, I trusted my helper”) and the Session Evaluation Scale (4 items, e.g., “I am glad I attended this session”). The internal consistency of this dimension of satisfaction in our sample was high (Cronbach α = 0.94).

Second, satisfaction with psychotherapists' interpersonal behaviors was evaluated after an evaluation of the perception of these behaviors. Clients initially had to evaluate their perception of psychotherapists' behaviors (see Manipulation Checks section), on the basis of eight adjectives from the IAS (Wiggins, 1995). The selected adjectives were representative of the two interpersonal dimensions of the circumplex model. For the communion dimension, these items were “gentlehearted,” “sympathetic,” “distant,” and “warmthless.” For the agency dimension, these items were “self-confident” “forceful” “shy” and “meek.” After evaluating their perception of these behaviors, participants then evaluated their level of satisfaction regarding each of these perceived behaviors on a Likert scale from extremely dissatisfied (level 1) to extremely satisfied (level 7). Since satisfaction is evaluated on participants' perception for each of these items, (e.g., “I perceive the therapist as highly sympathetic” and “I perceive the therapist as rather warmthless”), rather than on the items themselves, which could be conceptually opposite (e.g., sympathetic vs. warmthless), it is possible to evaluate the internal consistency of this dimension of satisfaction. It was high (Cronbach α = 0.88).

Third, the possibility of drop-out was evaluated by asking whether the client intended to pursue the psychotherapy after this first session on a Likert scale from totally disagree (1) to totally agree (5). So, the higher the score, the less the risk of drop-out.

Finally, to obtain a behavioral measure of satisfaction, participants had to specify how much they were prepared to pay the psychotherapist after this first session. To avoid influence from clients' socio-economic status, participants were free to choose the amount. To compare participants on this dependent variable, the given amounts for each psychotherapist were standardized for each participant on a Likert scale from 1 to 5, respectively representative of the lowest and highest amount of money that the participant was prepared to pay each psychotherapist.

### Manipulation check

To assess whether psychotherapists' behaviors shown in the videos were correctly perceived according to the interpersonal profiles expected, participants had to express their perception of the interpersonal profile for each psychotherapist presented in the different videos (see above).

## Results

### Manipulation checks

To check the experimental manipulation, clients' perceptions for each of the psychotherapist's interpersonal profiles were compared by means of repeated measures ANOVAs (see Table 1). The results indicated that participants perceived the interpersonal behaviors associated with the expected condition, for each of the five videos (Psychotherapists conditions). These results confirmed that the five videos were indeed representative of the five conditions corresponding to the psychotherapists' interpersonal profiles, namely Warm, Cold, Dominant, Submissive, and Neutral.

**Table 1:** Means (SDs in parentheses) of manipulation check (*N* = 75): perception of “psychotherapists' conditions” (within-subjects).

**Clients' perception**	**Psychotherapists' conditions**					***F***
	**Warm**	**Cold**	**Dominant**	**Submissive**	**Neutral**	
	***M (SD)***	***M (SD)***	***M (SD)***	***M (SD)***	***M (SD)***	
Warm	**8.49**^a^ **(1.50)**	2.81^d^ (1.31)	4.50^c^ (1.92)	7.48^b^ (1.61)	5.08^c^ (2.34)	129.88^***^
Cold	2.98^d^ (1.82)	**8.58**^a^ **(1.93)**	6.26^b^ (2.34)	4.57^c^ (2.00)	5.94^b^ (2.55)	69.90^***^
Dominant	6.68^c^ (1.71)	7.52^b^ (1.52)	**8.58**^a^ **(1.10)**	3.30^d^ (1.39)	6.24^c^ (1.72)	128.72^***^
Submissive	6.04^b^ (1.51)	2.78^d^ (1.10)	3.01^d^ (1.08)	**8.66**^a^ **(1.44)**	4.97^c^ (1.51)	215.15^***^

*Means with different superscripts are significantly different, using Bonferroni post-hoc tests in order to adjust the significance level. This adjustment involves an α-value divided by 10 (i.e., the number of comparisons), for each dependent measures*.

*** *p < 0.001*.

### What is the impact of the therapist's interpersonal dimensions?

To test the first two hypotheses, which are that a client will be more satisfied by (1) a warm therapist rather than a cold one and (2) a dominant therapist rather than a submissive one, we used a repeated measures ANOVA on each of the four dependent variables, with the five therapists (i.e., the experimental conditions) as within variable. Multiple comparisons between conditions allowed to specifically test the hypotheses. The Bonferroni procedure was used since the observed significant level is then adjusted according to the number of comparisons tested. In our design, the α-value of 0.05 is divided by 10. The results are presented in Table 2. Globally, they show highly significant effects of experimental condition for each of the four dependent variables: general satisfaction [*F*_(4, 270)_ = 33.87, *p* < 0.001, η2 = 0.314], behavior satisfaction [*F*_(3, 253)_ = 35.50, *p* < 0.001, η2 = 0.324], the drop-out measure [*F*_(4, 270)_ = 29.27, *p* < 0.001, η2 = 0.283] and the behavioral measure “money” [*F*_(4, 257)_ = 28.46, *p* < 0.01, η2 = 0.278]. Effect sizes indicate that about 30% of the variance in satisfaction scores are explained by the psychotherapist's interpersonal style.

**Table 2:** Simple effects of “psychotherapists' conditions” (within-subjects), for each satisfaction measure.

**Clients' profile**	**Measures of client satisfaction**	**Psychotherapists' Conditions**					**Psychotherapists' condition effect: *F***	**η^2^**	***df***
		**Warm**	**Cold**	**Dominant**	**Submissive**	**Neutral**			
		***M* (*SD*)**	***M* (*SD*)**	***M* (*SD*)**	***M* (*SD)***	***M* (*SD*)**			
Total Sample (*n* = 75)	General	30.32^a^ (0.86)	18.04^d^ (0.90)	24.84^b^ (0.98)	18.38^c,d^ (0.83)	21.61^b,c^ (0.91)	33.87^***^	0.314	(4,270)
	Behavior	40.53^a^ (1.10)	25.65^c^ (1.07)	32.37^b^ (1.14)	25.94^c^ (0.69)	28.84^b,c^ (1.09)	35.50^***^	0.324	(3,253)
	Drop-Out	3.75^a^ (0.13)	1.97^c^ (0.14)	2.89^b^ (0.16)	1.92^c^ (0.12)	2.44^b,c^ (0.14)	29.27^***^	0.283	(4,270)
	Money	4.24^a^ (0.14)	2.09^c^ (0.17)	3.06^b^ (0.18)	2.01^c^ (0.14)	2.60^b,c^ (0.16)	28.46^***^	0.278	(4,257)

*Means with different superscripts are significantly different, using Bonferroni post-hoc tests in order to adjust the significance level. This adjustment involves an α-value divided by 10 (i.e., the number of comparisons), for each dependent measures. Dependent variables: (1) General satisfaction is the evaluation of the session that the client had just experienced; (2) Behavior satisfaction is the evaluation of the psychotherapist's interpersonal behaviors; (3) Drop-out (reversed score) is an evaluation of the intend to pursuit the therapy; (4) Money is a behavioral measure, using the amount the client is ready to give to the therapist*.

*** *p < 0.001*.

**Figure 3:**
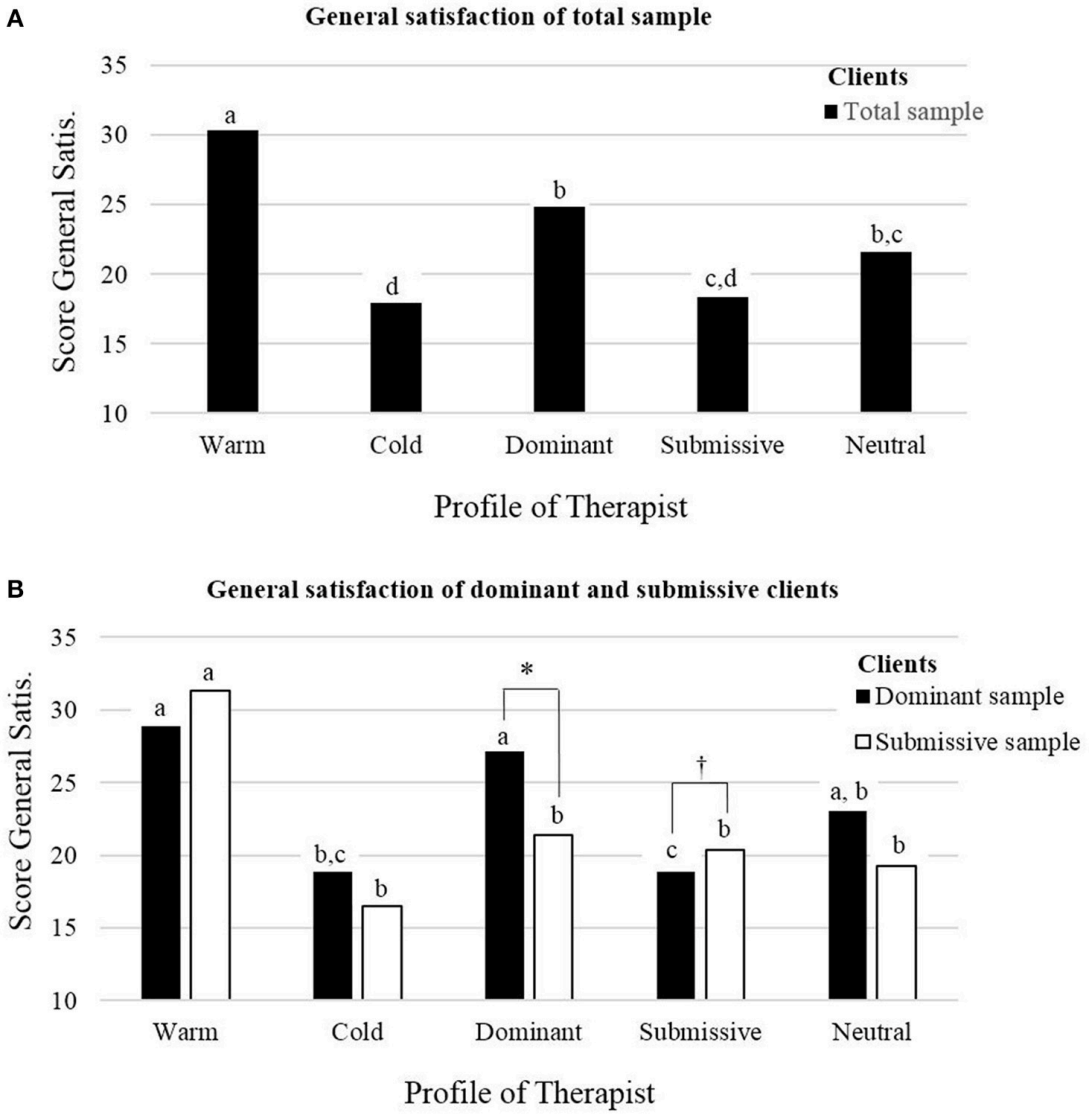
Impacts on the general satisfaction of the “psychotherapist's profile” (Within-Subjects)—**(A)** and the Interaction with “client's profile” (Between-Subjects)—**(B)**. **(A)** General satisfaction of total sample. **(B)** General satisfaction of dominant and submissive clients. Presentation of the general satisfaction across the five experimental conditions (i.e., therapist's interpersonal profiles) for the total sample (*n* = 75), for the dominant sample (*n* = 25) and for the submissive sample (*n* = 27). The **(A)** presents the effect of the psychotherapists' profile, illustrating hypotheses 1 and 2. The **(B)** presents the importance of taking into account the client's profile in order to see the interaction with the psychotherapist's profile, illustrating the hypothesis 3. Effect of the within-subjects variable in the case of interaction: means in a same sample with different superscripts are significantly different, using Bonferroni *post-hoc* tests in order to adjust the significance level. This adjustment involves an α-value divided by 10 (i.e., the number of comparisons). Effect of the between-subjects variable in the case of interaction: means across samples which are different are noted as: ^*†*^*p* < 0.1, ^*^p < 0.05.

Bonferroni *post-hoc* comparisons confirmed both the first and second hypothesis. Indeed, results first indicated that the Warm therapist was systematically and significantly more satisfying than the Cold therapist, irrespective of the dependent variable. To verify the specific effect size of the comparison between these two conditions, Cohen's d for paired means were calculated for each dependent variable. Large effect sizes were found for: (1) general satisfaction, *d* = 1.05, (2) behavior satisfaction, *d* = 0.99, (3) drop-out, *d* = 0.96, and (4) money, *d* = 0.96. Second, results indicated that clients were more satisfied by the Dominant therapist than the Submissive therapist, irrespective of dependent variable. Specific Cohen's ds of the comparison between these two conditions indicated medium effect sizes for the four dependent variables: (1) general satisfaction, *d* = 0.55, (2) behavior satisfaction, *d* = 0.54, (3) drop-out, *d* = 0.53 and (4) money, *d* = 0.48. Overall, Bonferroni comparisons systematically indicated that the Warm therapist was the most satisfying therapist across all experimental conditions, followed by the Dominant therapist and then by the Cold and Submissive therapists. The Neutral therapist was less satisfying than the Warm therapist but similar to the other conditions. To illustrate the effect of the psychotherapist's profile on client satisfaction, Figure 3A. presents general satisfaction across the five experimental conditions.

### What is the impact of the client's interpersonal dimensions?

With regard to our third hypothesis, we postulated that taking into account the client's interpersonal profile would allow to refine the results of the first and the second hypotheses although the best matching between therapist and client profiles remained exploratory. To test our hypothesis, for each dependent variable, repeated ANOVAs measures, with the five therapists as within-subjects variable and client's interpersonal dimensions as between-subjects variable were used on client's satisfaction scores. A first ANOVA was based on the client's communion profile (see Table 3A) while the second was based on the client's agency profile (see Table 3B).

**Table 3:** Simple effects and interactions of “psychotherapists' conditions” (within-subjects) and “clients' profile” (between-subjects) for each satisfaction measure.

**Measures of client satisfaction**	**Clients' profile**	**Psychotherapists' conditions**					**Psychotherapists' condition effect**		**Clients' profile effect**		**Interaction**	
		**Warm**	**Cold**	**Dominant**	**Submissive**	**Neutral**						
		***M* (*SD*)**	***M* (*S*D)**	***M* (*SD*)**	***M* (*SD*)**	***M* (*SD*)**	***F***	**η^2^**	***F***	**η^2^**	***F***	**η^2^**
**(A)**	**COMMUNION**											
General	Total	28.81^a^ (1.21)	18.62^c^ (1.17)	25.17^a,b^ (1.36)	18.47^c^ (1.11)	22.50^b,c^ (1.19)	14.23^***^	0.240	0.96	0.021	0.24	0.005
	Warm	27.76 (1.88)	18.64 (1.88)	25.17 (2.28)	17.70 (2.10)	21.29 (1.93)						
	Cold	29.87 (1.46)	18.60 (1.40)	25.16 (1.59)	19.23 (1.17)	23.70 (1.42)						
Behavior	Total	39.37^a^ (1.46)	26.73^c^ (1.45)	33.96^a,b^ (1.38)	26.29^c^ (0.96)	28.79^b,c^ (1.41)	17.32^***^	0.278	0.33	0.007	1.07	0.023
	Warm	39.88 (2.37)	27.35 (2.38)	34.35 (2.54)	25.64 (1.77)	26.11 (2.58)						
	Cold	38.86 (1.73)	26.10 (1.73)	33.56 (1.49)	26.93 (1.04)	31.46 (1.56)						
Drop-Out	Total	3.52^a^ (0.19)	2.01^c^ (0.19)	3.01^a,b^ (0.21)	1.91^c^ (0.16)	2.58^b,c^ (0.20)	12.63^***^	0.219	5.03^*^	0.101	0.03	0.001
	Warm	3.35 (0.27)	1.82 (0.30)	2.82 (0.37)	1.64 (0.24)	2.41 (0.29)						
	Cold	3.70 (0.24)	2.20 (0.24)	3.20 (0.23)	2.16 (0.19)	2.76 (0.25)						
Money	Total	4.03^a^ (0.20)	2.23^c^ (0.24)	3.44^a,b^ (0.25)	2.12^c^ (0.19)	2.89^b,c^ (0.22)	11.40^***^	0.202	0.01	0.000	0.35	0.008
	Warm	4.01 (0.34)	2.15 (0.39)	3.69(0.36)	2.01(0.34)	2.88 (0.40)						
	Cold	4.06 (0.23)	2.31 (0.29)	3.18(0.31)	2.22(0.22)	2.89 (0.24)						
**(B)**	**AGENCY**											
General	Total	30.09^a^ (1.14)	17.68^c^ (1.05)	24.26^b^ (1.09)	18.60^c^ (0.95)	21.17^b,c^ (1.03)	23.73^***^	0.322	1.39	0.027	3.81^**^	0.071
	Dominant	28.88 (1.97)	18.88 (1.59)	27.16 (1.48)	16.84 (1.21)	23.08 (1.48)						
	Submissive	31.29 (1.21)	16.48 (1.38)	21.37 (1.58)	20.37 (1.44)	19.25 (1.43)						
Behavior	Total	40.46^a^ (1.33)	25.33^c^ (1.26)	31.91^b^ (1.33)	26.22^c^ (0.78)	28.14^b,c^ (1.23)	25.57^***^	0.338	0.42	0.008	3.89^**^	0.072
	Dominant	38.16 (2.43)	27.12 (1.94)	34.60 (2.07)	24.00 (1.12)	29.88 (1.68)						
	Submissive	42.78 (1.21)	23.55 (1.62)	29.22 (1.70)	28.44 (1.10)	26.40 (1.78)						
Drop-Out	Total	3.74^a^ (0.17)	1.89^c^ (0.17)	2.84^b^ (0.16)	2.03^c^ (0.14)	2.30^b,c^ (0.17)	21.24^***^	0.298	1.06	0.021	3.09^*^	0.058
	Dominant	3.60 (0.31)	2.00 (0.28)	3.8 (0.20)	1.76 (0.18)	2.56 (0.26)						
	Submissive	3.88 (0.19)	1.77 (0.20)	2.40 (0.25)	2.29 (0.21)	2.03 (0.22)						
Money	Total	4.26^a^ (0.17)	2.01^b, c^ (0.20)	3.04^b^ (0.23)	2.15^c^ (0.15)	2.49^b,c^ (0.19)	20.95^***^	0.295	0.41	0.008	5.03^**^	0.091
	Dominant	3.78 (0.32)	2.23 (0.31)	3.43 (0.31)	1.57 (0.19)	2.70 (0.27)						
	Submissive	4.72 (0.13)	1.79 (0.28)	2.65 (0.33)	2.73 (0.23)	2.26 (0.26)						

*Means with different superscripts are significantly different, using Bonferroni post-hoc tests in order to adjust the significance level. This adjustment involves an α-value divided by 10 (i.e., the number of comparisons), for each dependent measures*.

*Dependent variables: (1) General satisfaction is the evaluation of the session that the client had just experienced; (2) Behavior satisfaction is the evaluation of the psychotherapist's interpersonal behaviors; (3) Drop-out (reversed score) is an evaluation of the intend to pursuit the therapy; (4) Money is a behavioral measure, using the amount the client is ready to give to the therapist*.

*Client Communion profile (n = 47): warm sample (n = 17) and cold sample (n = 30). Client Agency profile (n = 52): dominant sample (n = 25) and submissive sample (n = 27)*.

* *p < .05*.

** *p < 0.01*.

*** *p < 0.001*.

Globally (as can be seen in Table 3), previous results were confirmed and showed significant differences between psychotherapists' conditions across clients' interpersonal profiles, with large effects on the four measures of satisfaction varying from partial η2 = 0.202–0.338 meaning that 20.2–33.8% of the variance in satisfaction scores were explained by psychotherapists' interpersonal profile. Because results revealed a main effect of this within-subjects variable, Bonferroni *post-hoc* comparisons were used to determine the significant differences among psychotherapists' conditions (see superscripts in Table 3) for each dependent variable. In the reduced sample of the communion dimension (*N* = 47), it should be noted that the most satisfying therapist remained the Warm therapist which was significantly different from the Cold and Submissive therapists but not any more from the Dominant therapist. In the reduced sample of agency dimension (*N* = 52), the Warm therapist was significantly different from the others and was the most satisfying. The Dominant therapist was the second most appreciated one and he in turn was significantly different from the Cold and the Submissive therapists. The Neutral therapist was only different from the Warm therapist.

With regard to the communion dimension (warm vs. cold clients), in general, neither the main effect of the client's profile, nor the interaction were significant (see Table 3A). One exception was the significant client's profile main effect on drop-out, *F*_(1, 45)_ = 5.03, *p* = 0.030, η^2^ = 0.101. Intriguingly, examination of the means indicated that cold clients on average evaluated their intent to pursue therapy (*M* = 2.81 ± 0.11) as higher than warm clients (*M* = 2.41 ± 0.14). These general results indicate that whether clients were warm or cold they were not more satisfied by a specific profile of therapist. This is contradictory to both complementarity and similarity hypotheses found in the literature. The variation on the communion dimension does not determine client satisfaction according to the therapist profile (e.g., cold clients preferred the Warm therapist to the Cold therapist and warm clients were also more satisfied with the Warm therapist).

On the contrary, with regard to the agentic dimension (dominant vs. submissive clients), the results revealed different trends. Similar to previous findings, the main effect of client's agency profile was never significant on none of the variables (see Table 3B). However, with regard to the interactions between clients and therapists' profiles, results revealed that these interactions were systematically significant and explained between 6 and 9% of client satisfaction. To examine which variations explained the interaction, we first used Bonferroni *post-hoc* tests for each of the client's profiles (either dominant or submissive). Second, we also compared dominant and submissive client's evaluations on each psychotherapist condition using *t*-tests for independent samples. The results revealed that the significant interactions were mainly due to a higher satisfaction with the Dominant psychotherapist rated by dominant clients in comparison to submissive clients [for general satisfaction, *t*_(50)_ = 2.66, *p* = 0.010, satisfaction about the psychotherapist's behavior, *t*_(50)_ = 2.01, *p* = 0.049, intention to pursue the therapy, *t*_(50)_ = 2.70, *p* = 0.009, and finally the amount the client was ready to pay the therapist, *t*_(50)_ = 1.71, *p* = 0.093]. The reverse was true for submissive clients who rated the Submissive therapist as more satisfying than dominant clients [for general satisfaction, *t*_(50)_ = 1.85, *p* = 0.070, satisfaction about the psychotherapist's behavior, *t*_(50)_ = 2.82, *p* = 0.007, intention to pursue the therapy, *t*_(50)_ = 1.85, *p* = 0.069, and finally the amount the client was ready to pay the therapist, *t*_(50)_ = 3.81, *p* < 0.001]. Both dominant and submissive clients evaluated the Cold therapist similarly. For the Warm therapist, submissive clients tended to be more satisfied about the psychotherapist's behavior than dominant clients, *t*_(50)_ = 1.73, *p* = 0.089, and they intended to pay him more, *t*_(50)_ = 2.73, *p* = 0.009. These results seem to confirm the similarity hypothesis (i.e., dominant clients prefer a Dominant therapist while submissive clients prefer a Submissive therapist). However, this is only verified when using intergroup comparisons (i.e., dominant vs. submissive clients). To fully understand the significant interactions with clients' agency dimension, it is also necessary to consider the intragroup perspective and check which psychotherapist is preferred for both dominant and submissive clients. Bonferroni *post-hoc* tests indicated that submissive clients were only satisfied by the Warm therapist, and not by other psychotherapists' profiles, even if their satisfaction for the Submissive therapist was greater than that of dominant clients. On the contrary, dominant clients were generally satisfied by both the Dominant and the Warm therapists. Therefore, these results do not confirm either complementarity or similarity. They do however confirm that matching the client's agency profile with the therapist's profile is an additional determinant of client satisfaction. To illustrate this, Figure 3B. presents the general satisfaction of dominant and submissive clients for the five experimental conditions.

In sum, these last results indicate that taking clients' interpersonal profiles into account is a useful determinant of their satisfaction. It is however not possible to confirm either the complementarity, or the similarity hypothesis. Indeed, sometimes both were verified. Warm clients were more satisfied by a Warm therapist, which is concordant with the two hypotheses. In other cases, only the similarity hypothesis fitted the results. Indeed, dominant clients were more satisfied by the Dominant therapist than the Submissive one. But the fact that these clients were also satisfied by the Warm therapist corresponds to neither the similarity, nor the complementarity perspective. Finally, sometimes none of these hypotheses were verified since submissive clients were satisfied neither by the Dominant therapist (i.e., complementarity perspective), nor by the Submissive therapist (i.e., similarity perspective).

## Discussion

The present study highlights the importance of considering the therapist's interpersonal behaviors in order to predict client satisfaction during a first therapy session. In confirmation of the first hypothesis, the psychotherapist's communion dimension is a major explanatory variable of client satisfaction. Therapists' communion behaviors have a large effect on client satisfaction. Our study show that the Warm therapist is evaluated by clients as the most satisfactory, both by subjective and behavioral measures. These results are consistent with the broader literature on the efficacy of psychotherapy (e.g., Bozarth, 2007; Knox and Cooper, 2010; Farber and Doolin, 2011). They indicate that the therapist's communal and affiliative behaviors, i.e., his or her warmth, are essential to create a good and satisfying relationship at the very start of the psychotherapeutic process. Such behaviors may decrease the probability of dropping out from therapy after a first session. Since it is known that the therapist's warmth increases the therapeutic alliance that takes place over therapy sessions (Ackerman and Hilsenroth, 2003), and that client satisfaction and therapeutic alliance are related variables (Roos and Werbart, 2013), future studies should investigate how these variables are playing out together. For example, future longitudinal research could examine whether the effect of therapist's warmth on therapeutic alliance is mediated by the client satisfaction or if the therapist's warmth increases the therapeutic alliance, which then leads to higher client satisfaction.

With regard to the therapist's agentic behaviors, the present results also confirm the second hypothesis. Indeed, an agentic therapist is evaluated as being more satisfying than a submissive therapist. This means that overall, a more active therapist is preferred to a nonassertive one. Even if the Warm therapist is preferred over the Dominant therapist, our study highlights the importance of considering the psychotherapist's agentic characteristics to predict client satisfaction. Despite the fact that the literature is not completely consistent about the necessity for therapists to be directive or nondirective (Cain, 2013), our results seem to indicate that a therapist should show some assertive, rather than nonassertive, behaviors during a first session to increase client satisfaction.

For the first time, our innovative lab study allowed to take account of the interpersonal profile of the client and its match with several manipulated versions of a therapist profile. The results showed that taking the interaction between psychotherapist and client profiles into account (specifically their agency profile), significantly added about 7% of variance in explaining client satisfaction. Our results thus confirm the third hypothesis. These results fit with both the interpersonal (Kiesler, 1996) and the joint action perspectives (Knoblich et al., 2011) which postulate there should be better and worse matchings when two persons are interacting. Specifically, our results suggest that psychotherapists should adapt their level of agency depending on the client they have in front of them to increase client satisfaction. Dominant clients were more satisfied by a Dominant therapist than submissive clients. On the contrary, even if submissive clients were more satisfied by a Submissive therapist than dominant clients, they were mainly satisfied by a Warm therapist and not by others. Dominant clients favored the Dominant psychotherapist as much as the Warm psychotherapist, both in terms of satisfaction about the psychotherapist's behaviors and in terms of their overall satisfaction with the session they had just experienced.

With regard to previous interpersonal literature, these results suggest that the complementarity hypothesis does not hold concerning client satisfaction during a first session. In studying interactions between psychotherapist and client profiles, this research partially confirms the similarity hypothesis. Indeed, it fits for dominant clients but not for submissive ones since the latter clearly prefer a Warm therapist. Why does the agency dimension not work in a parallel way for dominant and submissive clients, which would lead to full confirmation of the similarity hypothesis? Three potential explanations are provided.

The first explanation is clinical and is centered on the potential needs of the clients. Indeed, individuals' interpersonal behaviors seem to be linked to their underlying motivations (Horowitz et al., 2006). It has been shown that, on one hand, dominant individuals tend to want to be autonomous in daily life, keeping control with regard to their environment (Fournier et al., 2011). Thus, a dominant psychotherapist, by using psycho-education and giving advice about dominant clients' situation, may give clients the feeling that they will be able to manage this situation themselves by recovering control. Thanks to this, such clients are likely to be satisfied with the session. On the other hand, submissive individuals tend to have a “fearful” attachment style (Horowitz et al., 1993), characterized by a need for reassurance about themselves and others. Accordingly, a warm psychotherapist, with an attitude of acceptance, listening and respect for the client's rhythm, can meet this need for reassurance. The fact that submissive clients were significantly more satisfied by a Warm psychotherapist compared to the four other styles of psychotherapist, including the complementary and the similar one, underlines the importance of the warm attitude with such clients. This first explanation suggests that it is important for the psychotherapist to detect the client's needs to increase client satisfaction, rather than adopting a complementary or a similar behavior.

The second explanation is conceptual and questions the structure of the agency dimension itself. According to Moskowitz (2005), the agency dimension should not be conceptualized as a bipolar dimension whose ends are dominant behaviors on one side and submissive behaviors on the other. Instead, Moskowitz (2005) hypothesized the existence of two unipolar dimensions working separately. One represents dominant behaviors while the other represents submissive behaviors. Therefore, Moskowitz has questioned both complementarity and similarity concepts as postulated in our hypotheses. The independence of two unipolar dimensions could explain why our results do not show strictly opposite effects between the two inverted poles of the agency dimension. If the agency dimension is made up of two unipolar dimensions working independently, this could explain the nonequivalence between dominant and submissive clients' satisfaction for dominant and submissive therapists (i.e., neither similar, nor opposite effect) and why dominant clients were satisfied by the dominant therapist, while submissive clients functioned differently and mainly preferred a warm psychotherapist.

The third explanation is methodological and raises questions about one of the experimental conditions. A truly submissive psychotherapist may not be very frequent in a clinical setting. The experimental condition “Submissive psychotherapist” was based on different behaviors characterized as submissive. For instance, among these different behaviors, the actor who played the submissive psychotherapist had to speak quietly (Moskowitz, 1994), show signs of embarrassment (Horowitz et al., 2003) or fidget with objects such as a pen or a writing pad (Gifford, 1991). In this context, it is possible that participating “clients” meeting the psychotherapist for the first time might find this type of behavior unsettling; they would consequently evaluate the psychotherapeutic experience as unsatisfactory. This means that the two extremities of the agency dimension were possibly not the most realistic application of the levels of control and autonomy taken by psychotherapists in the psychotherapeutic situation. The concept of nondirectiveness, as presented in the Rogerian conception (e.g., Grant, 1990), which is close to the concept of agency (Cooper and Norcross, 2016), may be more suited to the psychotherapeutic setting. Indeed, while from a conceptual viewpoint, the behaviors of directiveness and dominance are similar, nondirective behaviors, even if they are opposite to directive behaviors, were not included in the submissive manipulation of the agency dimension. It would perhaps be more realistic to meet a nondirective psychotherapist who would not necessarily show signs of embarrassment. To test this hypothesis, a future study should examine the influence of directive rather than dominant behaviors and of nondirective rather than submissive behaviors on client satisfaction.

Several limitations should also be underlined. First, considering that our interest is based on personality variables, our sample size was rather small. Indeed, for studies in personality psychology, the chance of finding a significant effect is reduced given the usually small effect size of personality variables (Roberts et al., 2007). This is particularly true in our study because of the *a posteriori* categorization based on four groups. However, the significant sizes of the obtained effects lead us to postulate that our results are interpretable and are worthy of consideration. A second limitation regards the level of ecological validity of the study. The simulated lab methodology used precluded taking account of the full complexity of the clinical reality. However, even if the use of videos may seem artificial, this methodology has previously been used to simulate real situations of interactions (Gaba, 2004) in several clinical settings such as clinical training (e.g., Anderson et al., 2016), observation of physicians' affiliative behaviors (Cousin and Schmid Mast, 2013) or the treatment of different disorders with virtual reality applications (e.g., Anderson et al., 2003). To ensure that participants could imagine being a potential client in the therapeutic situation as in our design, we used different open-ended questions during the debriefing at the end of the experiment. The questions were, for instance “Were the therapists realistic for you?” “Was it easy to imagine yourself as the patient?” “Were the sentences pronounced by the therapist or the patient appropriate for you?” Even if we have no quantitative data to show, it seems that the clinical conditioning was appropriate for the participants. The majority of participants reported positive impressions about the realism of the situation. Finally, a third limitation, linked to the previous one, is that the participants, as clients, had no possibility to answer spontaneously during their interaction with the psychotherapist. Consequently, participants could not show — and we could not examine *per se*—the complementarity “act-by-act,” meaning a behavior that subsequently leads to another behavior (Tracey, 2004; Sadler et al., 2011). Although the fictitious interactions spoken by the client were as neutral as possible, it is likely that participants would have reacted differently in a real-life situation. Therefore, our results should be considered from a dispositional rather than a situational point of view. In other words, they need to be understood according to the participant's disposition toward one or another interpersonal trait and not according to their capacities to adapt their interpersonal behaviors during a specific situation.

These two last limitations are linked to the lab methodology used. However, the choice of this methodology was deliberate and aimed to fill in a gap in traditional empirical studies in the clinical field. To answer our research question “which psychotherapist's behaviors influence client satisfaction during a first session of psychotherapy?”, it appears necessary to isolate the main explanatory variables, that is the interpersonal behaviors. It is known that such behaviors participate in the quick phenomenon of impression formation (Fiske and Neuberg, 1990). They are shown to depend on the situation and notably on the interlocutor (Sadler et al., 2011). They thus need to be standardized across participants. The originality of the design of this study allows to test a clinical phenomenon in an experimental situation, thus providing control over many alternative parameters in play. Moreover, the lab context is a great opportunity to present a client with several psychotherapists differing only in their interpersonal behaviors. This allows to obtain a fully controlled comparison of different therapists' behaviors. Of course, this study is a first step in understanding how interpersonal dimensions impact client satisfaction. These encouraging results should be replicated in more ecologically valid clinical situations.

In conclusion, this study showed that, by using an interpersonal perspective, it is possible to better understand the clinical phenomenon of client satisfaction during a first therapy session. The results suggest several consequences for therapists and their training. First, it is important for psychotherapists to be warm, including with clients who have a cold interpersonal style (Cousin et al., 2013). This confirms previous psychotherapy literature also on the role of warmth and the therapeutic alliance in the long run (e.g., Castonguay et al., 2011). Second, psychotherapists need to modulate their agentic style according to their clients' interpersonal style. For example, psychotherapists could increase their agentic behaviors with clients who are themselves agentic, while they should refrain from being too assertive and show warmth and full respect with unassertive clients. Third, if the results were confirmed in a more ecological context, this could also suggest that clinicians should be trained to identify their own and their client's interpersonal style as well as modulate their interpersonal behaviors according to their clients' preferences. A large literature shows that it is possible to train one's interpersonal communication or interpersonal skills (for a review, see Knapp and Daly, 2011). As in other fields (such as marketing, medical interventions, business community), it was shown that psychotherapists can be trained to better adapt their interpersonal behaviors to their clients and thus to avoid showing rigid behaviors which are detrimental to the therapeutic relationship (Tracey, 2005). Before being able to change behaviors, it is first necessary to better know ourselves and the behaviors that we preferentially set up. Psychotherapists' congruence (i.e., the experience of being fully self-aware), whose benefits have been demonstrated in the literature (e.g., Cornelius-White, 2007; Kolden et al., 2011), appears all important here. For psychotherapists, awareness of their own interpersonal style and favorite behaviors is a first step toward more flexibility. This flexibility should lead to suitable modulation of interpersonal behaviors (Erickson et al., 2009), resulting in the possibility of increasing client satisfaction after the first session and thereby decreasing drop-out risk and increasing the therapeutic alliance.

## Ethics statement

This study was carried out in accordance with the recommendations of “APA ethical standards, Ethics Commission of the Psychological Sciences Research Institute (Université catholique de Louvain)” with written informed consent from all subjects. All subjects gave written informed consent in accordance with the Declaration of Helsinki. The protocol was approved by the “Ethics Commission of the Psychological Sciences Research Institute (Université catholique de Louvain).”

## Author contributions

FM and EZ conceived and designed research. FM collected data. FM and EZ analyzed and interpreted data. FM produced the first draft of the final manuscript. FM and EZ written the final version of the manuscript. All authors approved the version to be published.

### Conflict of interest statement

The authors declare that the research was conducted in the absence of any commercial or financial relationships that could be construed as a potential conflict of interest.
